# Supplementary material for: Comparative genomics explains the evolutionary success of reef-forming corals
Source: eLife. 2016 May 24;5:e13288. doi: 10.7554/eLife.13288 (PMC4878875; doi:10.7554/eLife.13288)
Supplement: Figure 1—source data 1. — DOI: http://dx.doi.org/10.7554/eLife.13288.004 [file elife-13288-fig1-data1.docx]

**Figure 1-Source data 1.** Genomic data compiled in this study and their attributes.

| **Species** | **Reference** | **Resource** | **Platform** | **# Sequencing Reads** | **Insert length** | **# contigs** | **length [min/max]** | **mean length** | **# proteins** | **length [min/max]** | **mean length** | **# clusters > 100 aa (after CD-HIT)*** | **mean length** |
| --- | --- | --- | --- | --- | --- | --- | --- | --- | --- | --- | --- | --- | --- |
| *Acropora digitifera* | (Shinzato et al. 2011) | NCBI SRA DRP000456 | Roche 454/Illumina GAIIx | 895 M | 200 bp - 20kbp | 24967 | 250/19622 | 1091 | 17133 | 99/6412 | 365 | 16977 | 367 |
| *Acropora hyacinthus* | (Barshis et al. 2013) | NCBI SRA SRP017155 | Illumina | 528 M | NA | 44236 | 250/6581 | 474 | 12172 | 99/744 | 152 | 11589 | 153 |
| *Acropora millepora* | (Moya et al. 2012) | NCBI SRA SRP010485 | Roche 454/Illumina GAIIx | 559 M | 99 bp - 188 bp | 56260 | 226/35256 | 1367 | 35205 | 99/9792 | 358 | 28463 | 366 |
| *Acropora palmata* | (Schwarz et al. 2008) | NCBI EST DR982333–DR988505 | Sanger ABI-3730 | 14 K | NA | 14647 | 68/4502 | 727 | 7832 | 99/701 | 189 | 7522 | 191 |
| *Acropora tenuis* | http://www.bio.utexas.edu/research/matz_lab/matzlab/Data.html | http://www.bio.utexas.edu/research/matz_lab/matzlab/Data.html | Roche 454 | NA | NA | 65278 | 250/4809 | 554 | 19969 | 99/1105 | 170 | 18419 | 170 |
| *Amphimedon queenslandica* | (Srivastava et al. 2010) | NCBI BP PRJNA66531 | Sanger ABI-3730 | 2.2 M | 3kb - 36 kbp | ** | ** | ** | 29883 | 5/15891 | 392 | 22699 | 466 |
| *Anemonia viridis* | (Sabourault et al. 2009) | NCBI GB FK719875 - FK759813 | Sanger ABI-3730 | 50 K | NA | 10575 | 101/3378 | 645 | 5113 | 99/841 | 193 | 3235 | 201 |
| *Anthopleura elegantissima* | (Kitchen et al. 2015) | NCBI SRA SRP063463 | Illumina HiSeq2000 | 30 M | 330bp - 550 bp | 145832 | 201/58996 | 857 | 71700 | 99/13591 | 347 | 28527 | 285 |
| *Astreopora* sp. | Gruber et al., unpublished data | http://comparative.reefgenomics.org | NA | NA | NA | 55796 | 201/14117 | 940 | 35491 | 99/4705 | 268 | 23921 | 294 |
| *Edwardsiella lineata* | (Stefanik et al. 2014) | http://cnidarians.bu.edu/EdwardBase/cgi-bin/stock.cgi | Illumina GAIIx | 376 M | NA | 84400 | 100/32122 | 482 | 23662 | 99/8598 | 315 | 22492 | 316 |
| *Ephydatia muelleri* | RNA-seq assembly provided by Daniel J. Richter, Jennyfer Mora and Scott A. Nichols (sa.nichols321@gmail.com, University of Denver). | http://compagen.org/datasets/EMUE_T-PEP_130911 | NA | NA | NA | 85971 | 201/32493 | 809 | 29154 | 49/10830 | 326 | 20551 | 432 |
| *Favia* sp. | (Pooyaei Mehr et al. 2013) | NCBI BP PRJNA176860 | Illumina GAIIx | 78 M | NA | 121159 | 251/9823 | 663 | 63363 | 99/3236 | 252 | 26627 | 232 |
| *Fungia scutaria* | (Kitchen et al. 2015) | NCBI SRA SRP063463 | Illumina HiSeq2000 | 21 M | 330bp - 550 bp | 157884 | 201/37748 | 902 | 77289 | 99/8439 | 340 | 28265 | 304 |
| *Gorgonia ventalina* | (Burge et al. 2013) | NCBI SRA SRP014886 | Illumina GAIIx | 70 M | NA | 90230 | 257/8345 | 964 | 79078 | 99/2748 | 295 | 31334 | 248 |
| *Hydra magnipapillata* | (Chapman et al. 2010) | NCBI BP PRJNA12876 | Sanger ABI-3730 | 9.3 M | 3kb - 10 kb | ** | ** | ** | 19845 | 17/1724 | 192 | 15239 | 223 |
| *Madracis auretenra* | http://people.oregonstate.edu/~meyere/data.html | http://people.oregonstate.edu/~meyere/data.html | Illumina | NA | NA | 192296 | 201/10557 | 574 | 62118 | 99/2927 | 222 | 42119 | 214 |
| *Mnemiopsis leidyi* | (Ryan et al. 2013) | NCBI SRA SRP014828 | Roche 454/ Illumina GAIIx | 14.1 M | 400 bp - 4 kb | 15752 | 151/963 | 670 | 9048 | 99/313 | 177 | 2183 | 200 |
| *Monosiga brevicollis* | (King et al. 2008) | NCBI BP PRJNA19045 | NA | 0.6 M | 2 kb - 40 kb | ** | ** | ** | 9196 | 49/17829 | 598 | 8971 | 608 |
| *Montastraea cavernosa* | (Kitchen et al. 2015) | NCBI SRA SRP063463 | Illumina HiSeq2000 | 26 M | 330bp - 550 bp | 222244 | 201/73708 | 997 | 120867 | 99/14453 | 378 | 39938 | 280 |
| *Orbicella faveolata* | (Schwarz et al. 2008) | NCBI EST EY021828–EY031784 | Sanger ABI-3730 | NA | NA | 11611 | 100/2410 | 738 | 6038 | 99/599 | 182 | 5565 | 184 |
| *Nematostella vectensis* | (Putnam et al. 2007) | NCBI BP PRJNA19965 | Sanger ABI-3730 | 5.9 M | 3 kb - 35 kb | ** | ** | ** | 24780 | 49/8745 | 335 | 20123 | 383 |
| *Oscarella carmela* | (Nichols et al. 2012) | NCBI SRA SRP033441 | Illumina GAIIx | 388 M | NA | 67767 | 100/107672 | 853 | 22108 | 99/13178 | 198 | 21711 | 200 |
| *Platygyra carnosus* | (Sun et al. 2013) | NCBI SRA SRP010342 | Illumina HiSeq2000 | 59.6 M | 200 bp | ** | ** | ** | 103146 | 20/4307 | 181 | 66449 | 226 |
| *Pleurobrachia pileus* | (Moroz et al. 2014) | NCBI SRA SRP001155 | Roche 454/ Illumina GAIIx + HiSeq 2000 + MiSeq | 1,158 M | NA | 5983 | 103/2753 | 861 | 5726 | 99/868 | 267 | 4629 | 271 |
| *Pocillopora damicornis* | (Traylor-Knowles et al. 2011) | http://cnidarians.bu.edu/PocilloporaBase/cgi-bin/pdamdata.cgi | Roche 454 | 1.1 M | NA | 67254 | 250/10512 | 873 | 29875 | 99/646 | 192 | 20509 | 195 |
| *Porites astreoides* | (Kenkel et al. 2013) | <http://www.bio.utexas.edu/research/matz_lab/matzlab/Data.html> | Roche 454 | 0.29 M | NA | 38498 | 250/8171 | 559 | 16550 | 99/2398 | 173 | 15755 | 173 |
| *Porites australiensis* | (Shinzato et al. 2014) | NDBI SRA DRP000939 | Illumina HiSeq2000 | 71 M | NA | 62412 | 301/54798 | 1471 | 53470 | 99/14601 | 397 | 19567 | 454 |
| *Porites lobata* | http://comparative.reefgenomics.org | http://comparative.reefgenomics.org | Illumina HiSeq2000 | 50 M | 180 bp | 52990 | 250/34867 | 879 | 27906 | 99/9514 | 253 | 21062 | 279 |
| *Pseudodiploria strigosa* | http://people.oregonstate.edu/~meyere/data.html | http://people.oregonstate.edu/~meyere/data.html | NA | NA | NA | 107924 | 201/10217 | 587 | 33416 | 99/3211 | 221 | 24345 | 215 |
| *Seriatopora hystrix* | (Kitchen et al. 2015) | NCBI SRA SRP063463 | Illumina HiSeq2000 | 27 M | 330bp - 550 bp | 214882 | 201/56757 | 696 | 104573 | 99/9243 | 259 | 27680 | 246 |
| *Seriatopora* sp. | (Drake et al. 2013) | http://comparative.reefgenomics.org | Illumina MiSeq | 31 M | 425bp - 950 bp | ** | ** | ** | 46420 | 1/6540 | 261 | 35409 | 303 |
| *Stylophora pistillata* | http://comparative.reefgenomics.org | http://comparative.reefgenomics.org | Illumina HiSeq2000 | 1000M | 200 bp - 11kbp | ** | ** | ** | 22025 | 99/10583 | 395 | 21810 | 398 |
| *Trichoplax adherens* | (Srivastava et al. 2008) | NCBI BP PRJNA30931 | NA | 1.2 M | 2 kb - 40 kb | ** | ** | ** | 11520 | 49/7710 | 453 | 11000 | 467 |
|  |  |  |  |  |  |  |  |  |  |  |  |  |  |

** From genomic protein set

* Used for ortholog detection

**References**

Barshis DJ, Ladner JT, Oliver TA, Seneca FO, Traylor-Knowles N, Palumbi SR. 2013. Genomic basis for coral resilience to climate change. Proc. Natl. Acad. Sci. U.S.A. 110:1387-92.

Burge CA, Mouchka ME, Harvell CD, Roberts S. 2013. Immune response of the Caribbean sea fan, Gorgonia ventalina, exposed to an Aplanochytrium parasite as revealed by transcriptome sequencing. Front. Physiol. 4:180.

Chapman JA, Kirkness EF, Simakov O, Hampson SE, Mitros T, Weinmaier T, Rattei T, Balasubramanian PG, Borman J, Busam D, et al. 2010. The dynamic genome of Hydra. Nature. 464:592-6.

Drake JL, Mass T, Haramaty L, Zelzion E, Bhattacharya D, Falkowski PG. 2013. Proteomic analysis of skeletal organic matrix from the stony coral Stylophora pistillata. Proc. Natl. Acad. Sci. U.S.A. 110:3788-93.

Kenkel CD, Meyer E, Matz MV. 2013. Gene expression under chronic heat stress in populations of the mustard hill coral (Porites astreoides) from different thermal environments. Mol. Ecol. 22:4322-34.

King N, Westbrook MJ, Young SL, Kuo A, Abedin M, Chapman J, Fairclough S, Hellsten U, Isogai Y, Letunic I, et al. 2008. The genome of the choanoflagellate Monosiga brevicollis and the origin of metazoans. Nature. 451:783-8.

Kitchen SA, Crowder CM, Poole AZ, Weis VM, Meyer E. 2015. De Novo Assembly and Characterization of Four Anthozoan (Phylum Cnidaria) Transcriptomes. G3 (Bethesda). 5:2441-52

Moroz LL, Kocot KM, Citarella MR, Dosung S, Norekian TP, Povolotskaya IS, Grigorenko AP, Dailey C, Berezikov E, Buckley KM, et al. 2014. The ctenophore genome and the evolutionary origins of neural systems. Nature. 510:109-14.

Moya A, Huisman L, Ball EE, Hayward DC, Grasso LC, Chua CM, Woo HN, Gattuso JP, Foret S, Miller DJ. 2012. Whole transcriptome analysis of the coral Acropora millepora reveals complex responses to CO2-driven acidification during the initiation of calcification. Mol. Ecol. 21:2440-54.

Nichols SA, Roberts BW, Richter DJ, Fairclough SR, King N. 2012. Origin of metazoan cadherin diversity and the antiquity of the classical cadherin/beta-catenin complex. Proc. Natl. Acad. Sci. U.S.A. 109:13046-51.

Pooyaei Mehr SF, DeSalle R, Kao HT, Narechania A, Han Z, Tchernov D, Pieribone V, Gruber DF. 2013. Transcriptome deep-sequencing and clustering of expressed isoforms from Favia corals. BMC Genomics. 14:546.

Putnam NH, Srivastava M, Hellsten U, Dirks B, Chapman J, Salamov A, Terry A, Shapiro H, Lindquist E, Kapitonov VV, et al. 2007. Sea anemone genome reveals ancestral eumetazoan gene repertoire and genomic organization. Science. 317:86-94.

Ryan JF, Pang K, Schnitzler CE, Nguyen AD, Moreland RT, Simmons DK, Koch BJ, Francis WR, Havlak P, Smith SA, et al. 2013. The genome of the ctenophore Mnemiopsis leidyi and its implications for cell type evolution. Science. 342:1242592.

Sabourault C, Ganot P, Deleury E, Allemand D, Furla P. 2009. Comprehensive EST analysis of the symbiotic sea anemone, Anemonia viridis. BMC Genomics. 10:333.

Schwarz JA, Brokstein PB, Voolstra C, Terry AY, Manohar CF, Miller DJ, Szmant AM, Coffroth MA, Medina M. 2008. Coral life history and symbiosis: functional genomic resources for two reef building Caribbean corals, Acropora palmata and Montastraea faveolata. BMC Genomics. 9:97.

Shinzato C, Inoue M, Kusakabe M. 2014. A snapshot of a coral "holobiont": a transcriptome assembly of the scleractinian coral, porites, captures a wide variety of genes from both the host and symbiotic zooxanthellae. PLoS One. 9:e85182.

Shinzato C, Shoguchi E, Kawashima T, Hamada M, Hisata K, Tanaka M, Fujie M, Fujiwara M, Koyanagi R, Ikuta T, et al. 2011. Using the Acropora digitifera genome to understand coral responses to environmental change. Nature. 476:320-3.

Srivastava M, Begovic E, Chapman J, Putnam NH, Hellsten U, Kawashima T, Kuo A, Mitros T, Salamov A, Carpenter ML, et al. 2008. The Trichoplax genome and the nature of placozoans. Nature. 454:955-60.

Srivastava M, Simakov O, Chapman J, Fahey B, Gauthier ME, Mitros T, Richards GS, Conaco C, Dacre M, Hellsten U, et al. 2010. The Amphimedon queenslandica genome and the evolution of animal complexity. Nature. 466:720-6.

Stefanik DJ, Lubinski TJ, Granger BR, Byrd AL, Reitzel AM, DeFilippo L, Lorenc A, Finnerty JR. 2014. Production of a reference transcriptome and transcriptomic database (EdwardsiellaBase) for the lined sea anemone, Edwardsiella lineata, a parasitic cnidarian. BMC Genomics. 15:71.

Sun J, Chen Q, Lun JC, Xu J, Qiu JW. 2013. PcarnBase: development of a transcriptomic database for the brain coral Platygyra carnosus. Mar. Biotechnol. 15:244-51.

Traylor-Knowles N, Granger BR, Lubinski TJ, Parikh JR, Garamszegi S, Xia Y, Marto JA, Kaufman L, Finnerty JR. 2011. Production of a reference transcriptome and transcriptomic database (PocilloporaBase) for the cauliflower coral, Pocillopora damicornis. BMC Genomics. 12:585.
