# Supplementary material for: Comparative genomics explains the evolutionary success of reef-forming corals
Source: eLife. 2016 May 24;5:e13288. doi: 10.7554/eLife.13288 (PMC4878875; doi:10.7554/eLife.13288)
Supplement: Figure 2—source data 1. — DOI: http://dx.doi.org/10.7554/eLife.13288.006 [file elife-13288-fig2-data1.docx]

**Figure 2-Source data 1.** Major components of the human ion trafficking system identified in the coral genomic data. ‘X’ indicates the presence of genes in the corresponding species. ‘P’ indicates pseudogene.

| Species | TRPA1 | TRPV1 | TRPV2 | TRPV3 | TRPV4 | TRPV5 | TRPV6 | TRPM1 | TRPM2 | TRPM3 | TRPM4 | TRPM5 | TRPM6 | TRPM7 | TRPM8 | TRPC1 | TRPC2 | TRPC3 | TRPC4 | TRPC5 | TRPC6 | TRPC7 | ASIC1 | ASIC2 | ASIC3 | ASIC4 | ASIC5 | ORAI1 | ORAI2 | ORAI3 | ITPR1 | ITPR2 | ITPR3 | PLCG1 | PLCG2 | PLCB | PLCD | PLCH | NFAT5 | Calmodulin | CLCN1 | CLCN2 | CLCN6 | KCNJ2 | KCNJ3 | KCNJ4 | KCNJ5 | KCNJ6 | KCNJ8 | KCNJ9 | KCNJ12 | KCNJ18 |
| --- | --- | --- | --- | --- | --- | --- | --- | --- | --- | --- | --- | --- | --- | --- | --- | --- | --- | --- | --- | --- | --- | --- | --- | --- | --- | --- | --- | --- | --- | --- | --- | --- | --- | --- | --- | --- | --- | --- | --- | --- | --- | --- | --- | --- | --- | --- | --- | --- | --- | --- | --- | --- |
| *Acropora digitifera* | X |  |  |  |  |  | X |  |  | X |  |  |  |  |  |  | p |  | X | X | X | X | X | X | X |  |  | X |  |  | X |  |  | X |  |  | X | X |  | X |  | X |  | X |  |  |  |  |  |  | X |  |
| *Acropora hyacinthus* | X |  |  |  |  |  |  |  | X |  |  |  |  |  |  |  | P |  |  |  |  |  |  |  |  |  |  |  |  |  | X |  |  |  | X |  |  |  |  | X | X |  |  |  |  |  |  |  |  |  |  |  |
| *Acropora millepora* | X |  |  |  |  |  | X |  |  | X |  |  | X | X |  |  | p |  | X | X | X | X |  | X | X |  |  | X |  |  | X |  |  | X |  | X | X | X | X | X |  | X |  | X |  |  |  | X |  |  | X |  |
| *Acropora palmata* | X |  |  |  |  |  |  |  | X |  |  |  |  |  |  |  | P |  |  |  |  | X |  |  |  |  |  |  |  |  | X |  |  |  |  |  |  |  |  | X |  |  |  |  |  |  |  |  |  |  |  |  |
| *Acropora tenuis* | X | X |  | X |  |  |  |  | X | X |  |  | X |  |  |  | p |  | X | X | X |  | X | X | X |  |  |  |  |  | X |  |  | X | X |  | X |  | X | X |  |  |  |  |  |  |  |  |  |  | X |  |
| *Astreopora sp.* | X |  |  |  |  |  | X | X |  | X |  |  |  |  |  |  | P |  | X |  |  | X | X | X |  |  |  |  |  |  | X |  |  | X |  |  | X |  | X | X |  | X |  | X |  |  |  | X | X |  |  |  |
| *Favia sp.* | X |  |  |  |  |  | X |  | X | X |  |  |  | X |  |  | p |  | X |  | X |  |  | X |  |  |  | X |  |  | X |  |  | X |  | X | X | X | X | X |  | X |  |  |  |  |  |  |  |  |  |  |
| *Fungia scutaria* | X | X |  |  |  | X | X | X |  | X |  |  | X | X |  |  | P | X | X | X |  |  | X | X | X |  |  | X |  |  | X |  |  | X |  | X | X | X | X | X |  | X |  |  | X |  |  |  | X |  |  |  |
| *Madracis auretenra* | X |  |  |  |  |  | X |  |  | X |  |  |  |  |  |  | p |  | X | X | X |  | X | X | X |  |  | X |  |  | X |  |  | X |  |  |  | X | X | X |  | X |  | X |  |  |  | X |  | X | X |  |
| *Montastraea cavernosa* | X |  |  |  |  |  | X | X |  | X |  |  |  |  |  |  | P | X | X | X | X |  | X | X | X |  |  | X |  |  | X |  |  | X |  | X | X | X | X | X |  | X |  |  |  |  |  |  | X |  |  | X |
| *Montastraea faveolata* | X |  |  |  |  | X |  |  |  |  |  |  |  |  |  |  | p |  |  |  |  |  |  |  |  |  |  |  |  |  | X |  |  |  |  |  |  |  | X | X |  |  |  |  |  |  |  |  |  |  |  |  |
| *Platygyra carnosus* | X |  |  |  |  |  |  | X | X | X |  |  |  |  |  |  | P |  | X | X |  | X |  |  |  |  |  |  |  |  | X |  |  | X |  | X |  |  | X | X |  | X |  | X |  |  |  |  | X |  |  |  |
| *Pocillopora damicornis* | X |  |  |  | X |  |  |  |  | X |  |  |  |  |  |  | p |  | X | X | X |  | X | X |  |  | X |  |  |  |  | X |  | X | X |  |  | X | X | X |  |  |  |  |  |  |  |  |  | X | X |  |
| *Porites astreoides* | X |  |  |  |  |  |  |  |  | X |  |  |  |  |  |  | P |  |  | X | X |  |  |  |  |  | X |  |  |  | X |  |  |  |  |  |  | X | X | X |  |  |  | X |  |  |  |  |  |  |  |  |
| *Porites australiensis* | X |  |  |  |  | X |  | X |  | X |  |  | X | X |  |  | p | X | X | X | X | X |  | X | X |  |  | X |  |  | X |  |  | X |  | X | X |  | X | X |  | X | X | X |  | X | X |  |  |  |  |  |
| *Porites lobata* | X |  |  |  |  |  |  | X | X | X |  | X |  | X |  |  | P |  | X |  |  | X |  | X | X |  | X | X |  |  | X |  |  | X |  |  |  |  | X | X | X | X |  | X |  | X |  |  |  |  |  |  |
| *Pseudodiploria strigosa* | X |  |  |  | X |  | X | X | X | X |  |  |  |  |  |  | p |  |  |  | X |  | X | X | X |  | X | X |  |  | X | X |  |  |  |  |  |  | X | X |  | X |  | X |  |  |  | X | X |  |  |  |
| *Seriatopora hystrix* | X |  |  |  | X |  | X | X | X | X |  |  | X | X |  |  | P | X | X | X | X | X | X |  | X |  |  |  |  |  | X | X |  | X |  | X |  |  | X | X |  | X |  |  |  | X |  |  |  |  |  |  |
| *Seriatopora sp.* | X |  |  |  | X |  |  |  | X | X |  | X |  | X |  |  | p |  | X | X | X |  | X | X | X |  |  | X |  | X | X |  |  | X | X |  |  | X | X | X | X |  |  |  |  |  | X |  |  |  | X |  |
| *Stylophora pistillata* | X |  |  |  |  |  | X |  |  | X |  |  |  |  |  |  | P |  | X | X | X |  | X | X | X |  | X | X |  |  | X |  |  | X |  | X | X | X | X | X |  | X |  | X |  |  |  |  |  | X |  |  |

| Species | SCNN1A | SCNN1B | SCNN1G | SLC9C1 | SLC9C2 | ATP1A1 | ATP1A2 | ATP1A3 | SLC24.1 | SLC24.2 | SLC24.3 | SLC24.4 | SLC24.5 | SLC4A1 | SLC4A2 | SLC4A7 | SLC4A8 | SLC4A10 | AQP4 | MCU | VDAC1 | VDAC2 | VDAC3 | MCOLN1 | MCOLN2 | MCOLN3 | TPC1 | TPC2 | PPP3C A | PPP3C B | PPP3C C | GJ A1 | GJ B2 | GJ C3 | GNAQ | GNA11 | GNAI1 | GNAS | GNAO1 | GNA12 | GNA13 | GNAT | PRKCA | NFKB |
| --- | --- | --- | --- | --- | --- | --- | --- | --- | --- | --- | --- | --- | --- | --- | --- | --- | --- | --- | --- | --- | --- | --- | --- | --- | --- | --- | --- | --- | --- | --- | --- | --- | --- | --- | --- | --- | --- | --- | --- | --- | --- | --- | --- | --- |
| *Acropora digitifera* | X | X |  |  |  | X |  |  |  | X | X | X |  |  | X |  | X |  | X | X |  | X |  |  | X | X | X | X |  | X | X |  |  |  |  |  | X | X | X |  | X |  | X | X |
| *Acropora hyacinthus* |  |  |  |  |  |  |  |  |  | X |  |  |  |  |  |  |  |  |  | X | X |  |  |  |  | X | X |  |  | X | X |  |  |  | X | X | X |  | X |  |  |  |  |  |
| *Acropora millepora* | X | X |  |  |  | X |  | X |  | X |  | X | X |  | X |  |  | X | X | X |  | X |  |  |  | X | X | X | X | X |  |  |  |  | X |  | X | X | X |  | X | X | X | X |
| *Acropora palmata* |  |  |  |  |  |  |  |  |  | X | X | X |  |  |  |  |  |  |  | X | X |  |  |  |  |  | X |  |  |  |  |  |  |  | X |  | X |  | X |  |  |  |  |  |
| *Acropora tenuis* |  |  |  |  |  | X |  |  | X | X |  | X |  |  | X |  |  |  |  | X |  | X |  |  |  | X | X |  |  | X | X |  |  |  | X |  | X |  | X |  |  |  |  | X |
| *Astreopora sp.* |  |  |  | X |  |  | X | X |  | X |  | X |  |  | X | X |  | X | X | X |  | X |  |  | X | X | X |  | X | X |  |  |  |  | X |  | X | X | X |  | X |  | X | X |
| *Favia sp.* |  |  |  |  |  | X | X | X |  | X | X | X |  | X | X |  |  | X |  |  | X | X | X |  |  | X | X |  | X |  | X |  |  |  | X |  | X | X |  | X | X |  | X | X |
| *Fungia scutaria* | X | X |  | X |  | X | X | X |  | X |  | X | X |  | X |  |  |  | X | X |  | X |  |  | X | X | X |  | X |  |  |  |  |  | X |  | X | X | X |  | X |  | X | X |
| *Madracis auretenra* |  |  |  |  |  |  | X | X |  | X | X |  | X | X |  |  |  | X | X | X | X | X |  | X | X | X | X |  | X |  | X |  | X |  | X | X | X | X | X |  | X |  | X | X |
| *Montastraea cavernosa* | X | X |  |  |  | X | X | X |  | X | X | X | X |  | X |  | X |  | X | X |  | X |  |  | X | X | X |  | X |  |  |  |  |  | X |  | X | X | X |  | X |  | X | X |
| *Montastraea faveolata* | X |  |  |  |  |  |  |  |  | X |  |  |  |  |  |  |  |  |  |  |  | X |  |  | X |  | X |  |  |  |  |  |  |  |  |  | X |  |  |  | X |  |  | X |
| *Platygyra carnosus* | X |  |  |  |  |  | X | X |  | X |  |  |  |  | X |  |  | X |  | X |  | X |  |  | X | X | X |  | X |  | X |  |  |  | X |  | X | X | X |  | X |  |  | X |
| *Pocillopora damicornis* | X |  |  |  |  | X |  |  |  | X |  |  |  |  |  |  |  | X | X | X |  | X |  |  | X | X | X |  | X |  |  | X |  |  | X | X | X |  | X |  |  | X |  |  |
| *Porites astreoides* |  |  |  |  |  |  | X | X |  | X | X |  |  |  | X |  | X |  |  | X | X |  |  |  | X | X | X |  | X |  |  |  |  |  | X |  | X |  |  |  |  |  |  | X |
| *Porites australiensis* | X |  | X |  |  |  | X | X |  | X |  | X | X |  | X |  | X | X | X | X | X |  |  |  | X | X | X | X | X |  |  |  |  |  |  |  | X | X | X |  | X |  | X | X |
| *Porites lobata* |  |  |  |  |  |  |  |  |  | X |  |  |  |  | X |  |  | X | X | X | X |  |  |  | X | X | X |  | X | X |  |  |  |  | X |  | X |  | X |  | X |  | X | X |
| *Pseudodiploria strigosa* | X | X |  |  |  | X | X | X |  | X |  | X |  |  |  |  | X |  | X | X |  | X |  |  | X | X | X |  |  |  |  |  |  |  | X |  | X | X | X |  |  | X | X | X |
| *Seriatopora hystrix* | X |  |  |  |  |  |  | X |  | X |  |  |  | X | X |  | X | X | X | X |  | X |  |  | X |  | X |  | X |  |  |  |  | X | X |  | X | X | X | X |  |  | X | X |
| *Seriatopora sp.* | X |  |  | X |  | X |  | X |  | X |  | X | X | X | X |  | X |  |  | X |  | X |  |  | X |  |  |  | X |  |  |  |  | X | X |  | X |  | X |  | X |  | X | X |
| *Stylophora pistillata* | X | X |  |  | X | X |  | X |  | X |  | X | X |  | X |  | X |  | X | X | X |  |  |  | X | X | X | X | X | X |  |  |  | X | X |  | X | X | X |  | X |  | X | X |
