# Supplementary material for: Comparative genomics explains the evolutionary success of reef-forming corals
Source: eLife. 2016 May 24;5:e13288. doi: 10.7554/eLife.13288 (PMC4878875; doi:10.7554/eLife.13288)
Supplement: Supplementary file 1. — The values in parentheses show the number of taxa in which the gene sequence was recovered in the genomic database. DOI: http://dx.doi.org/10.7554/eLife.13288.022 [file elife-13288-supp1.docx]

**Supplement file 1**. Taxonomic compilation and presence/absence in each taxon for genes involved in oxidative stress, DNA repair, cell cycle and apoptosis. The values in parentheses show the number of taxa in which the gene sequence was recovered in the genomic database. The genes (if present) are indicated by ‘X’ for each species.

| Species | AIF (26/32) | APAF1 (31/32) | Atm (28/32) | Bax (28/32) | bct2 (28/32) | Cad (18/32) | caspase3 (30/32) | caspase8 (30/32) | caspase9 (30/32) | Catalase (29/32) | cdc2 (32/32) | cdc25 (22/32) | cip1/p21 (23/32) | Cytc (22/32) | Fas (22/32) | Gpx (32/32) | Hausp (31/32) | Icad (17/32) | mdm2 (20/32) | Mortalin (32/32) | Nos (30/32) | p53 (24/32) | Photolyase (2-/32) | Mnsod (30/32) | Cu/Zn sod (31/32) | Tnf (7/32) | topo2 (25/32) |
| --- | --- | --- | --- | --- | --- | --- | --- | --- | --- | --- | --- | --- | --- | --- | --- | --- | --- | --- | --- | --- | --- | --- | --- | --- | --- | --- | --- |
| *Trichoplax adherens* | X | X | X | X | X |  | X | X | X | X | X | X |  | X | X | X | X |  | X | X | X | X |  | X | X |  | X |
| *Monosiga brevicollis* | X |  |  |  |  |  |  |  |  | X | X |  |  | X |  | X | X |  |  | X | X |  |  | X | X |  |  |
| *Amphimedon queenslandica* | X | X | X | X | X |  | X | X | X | X | X | X |  | X | X | X | X |  |  | X | X | X | X | X | X |  | X |
| *Ephydatia muelleri* | X | X | X |  |  |  | X | X | X | X | X | X |  |  | X | X | X |  |  | X | X | X | X | X | X |  | X |
| *Oscarella carmela* |  | X | X | X | X |  | X | X | X | X | X |  |  |  | X | X | X |  | X | X | X |  |  | X |  |  | X |
| *Mnemiopsis leidyi* |  | X |  |  |  |  |  |  |  |  | X |  |  | X |  | X |  |  |  | X |  |  |  | X | X |  |  |
| *Pleurobrachia pileus* |  | X | X |  |  |  | X | X | X |  | X |  | X |  |  | X | X |  |  | X | X | X |  |  | X |  |  |
| *Hydra magnipapillata* | X | X | X | X | X |  | X | X | X | X | X | X |  | X |  | X | X |  |  | X | X |  |  | X | X |  | X |
| *Anemonia viridis* | X | X |  | X | X |  | X | X | X | X | X |  | X |  |  | X | X |  | X | X | X |  |  |  | X |  |  |
| *Anthopleura elegantissima* | X | X | X | X | X |  | X | X | X | X | X | X | X | X | X | X | X |  | X | X | X | X | X | X | X | X | X |
| *Nematostella vectensis* | X | X | X | X | X | X | X | X | X | X | X | X | X | X | X | X | X | X |  | X | X | X | X | X | X |  | X |
| *Gorgonia ventalina* | X | X | X | X | X | X | X | X | X | X | X | X | X | X | X | X | X | X | X | X | X | X | X | X | X |  | X |
| *Acropora digitifera* | X | X | X | X | X | X | X | X | X | X | X | X |  | X | X | X | X | X | X | X | X | X | X | X | X | X | X |
| *Acropora hyacinthus* | X | X | X | X | X | X | X | X | X | X | X |  | X | X | X | X | X | X |  | X | X |  | X | X | X |  | X |
| *Acropora millepora* | X | X | X | X | X | X | X | X | X | X | X | X | X | X | X | X | X | X | X | X | X | X | X | X | X | X | X |
| *Acropora palmata* |  | X | X | X | X | X | X | X | X |  | X | X |  | X |  | X | X | X | X | X |  |  |  | X | X |  |  |
| *Acropora tenuis* | X | X | X | X | X | X | X | X | X | X | X | X | x | X | X | X | X | X | X | X | X | X | X | X | X |  | X |
| *Astreopora sp.* | X | X | X | X | X | X | X | X | X | X | X | X | X |  | X | X | X | X | X | X | X | X | X | X | X | X | X |
| *Favia sp.* |  | X | X | X | X |  | X | X | X | X | X | X | X |  |  | X | X | X | X | X | X | X | X | X | X |  | X |
| *Fungia scutaria* | X | X | X | X | X | X | X | X | X | X | X | X | X | X |  | X | X | X | X | X | X | X | X | X | X |  | X |
| *Madracis auretenra* | X | X | X | X | X | X | X | X | X | X | X | X | X | X | X | X | X | X | X | X | X | X | X | X | X |  | X |
| *Montastraea cavernosa* | X | X | X | X | X |  | X | X | X | X | X | X | X | X | X | X | X |  | X | X | X | X | X | X | X |  | X |
| *Montastraea faveolata* |  | X |  | X | X | X | X | X | X | X | X |  | X | X |  | X | X |  |  | X | X |  |  | X | X |  |  |
| *Platygyra carnosus* | X | X | X | X | X | X | X | X | X | X | X | X | X | X | X | X | X | X | X | X | X | X | X | X | X |  | X |
| *Pocillopora damicornis* | X | X | X | X | X | X | X | X | X | X | X | X | X | X | X | X | X |  |  | X | X | X | X | X | X |  |  |
| *Porites astreoides* | X | X | X | X | X |  | X | X | X | X | X |  | X | X | X | X | X |  | X | X | X | X | X | X | X |  | X |
| *Porites australiensis* | X | X | X | X | X | X | X | X | X | X | X |  | X |  | X | X | X | X | X | X | X | X |  | X | X | X | X |
| *Porites lobata* | X | X | X | X | X | X | X | X | X | X | X |  | X |  | X | X | X | X | X | X | X | X |  | X | X | X | X |
| *Pseudodiploria strigosa* | X | X | X | X | X |  | X | X | X | X | X | X | X | X | X | X | X | X | X | X | X | X |  | X | X | X | X |
| *Stylophora pistillata* | X | X | X | X | X | X | X | X | X | X | X | X | X |  |  | X | X | X |  | X | X | X | X | X | X |  | X |
| *Seriatopora hystrix* | X | X | X | X | X | X | X | X | X | X | X | X | X |  | X | X | X | X | X | X | X | X | X | X | X |  | X |
| *Seriatopora sp.* | X | X | X | X | X | X | X | X | X | X | X | X | X | X | X | X | X |  |  | X | X | X | X | X | X |  | X |
